# AWJ Cutting Process Quality Modeling and Optimization Based on Footprint Angle

**DOI:** 10.3390/ma18245548

**Published:** 2025-12-10

**Authors:** Andrzej Perec, Elzbieta Kawecka, Wojciech Zajac

**Affiliations:** Faculty of Technology, The Jacob of Paradies University, 66-400 Gorzów Wielkopolski, Poland; ekawecka@ajp.edu.pl (E.K.); wzajac@ajp.edu.pl (W.Z.)

**Keywords:** abrasive water jet, machining efficiency, machining quality, modeling, prediction

## Abstract

**Highlights:**

**What are the main findings?**

- The cutting process circumstances evaluation by measuring the footprints angle and jet deflection angle.
- Quality control of the AWJ machining process was achieved by measuring the jet deflection angle.

**What is the implication of the main finding?**

- The possibility of setting cutting parameters for new materials not contained in the implemented model in the AWJ cutting machine.
- Establish the possibility of assessing the jet deflection angle and correcting actual machining conditions at the required efficiency while achieving the assumed surface quality.

**Abstract:**

Various materials may be machined using the abrasive water jet (AWJ) cutting method. Many control factors, such as abrasive flow, operating pressure, and traverse speed, influence the efficiency and surface quality of AWJ-cut components. The common distinguishing factor of process efficiency and quality is the angle of machining footprints (striation angle). This paper presents research results on the control parameters as a method of influencing the striation angle through the angle level of machining footprints to achieve high efficiency and quality, for example, the high-impact and abrasive-resistant steel. This will enable quality control of the AWJ cutting process by continuously measuring the jet deflection angle in an online mode and adjusting these parameters in real-time to maintain high efficiency and the required surface quality. The particular interest in utilizing this basis is the possibility of setting cutting parameters for new materials not included in the implemented model of the AWJ cutting machine.

## 1. Introduction

A high-velocity water jet, combined with abrasive grains, is used in the unconventional machining technique known as AWJ machining to treat materials. It is a versatile cold-cutting technology that uses a high-velocity waterjet (typically 600–900 m/s) loaded with hard abrasive particles (usually garnet) to erode virtually any engineering material without inducing thermal damage or significant mechanical stresses. It is renowned for its capacity to machine intricate forms without causing thermal damage. High-pressure AWJ processing has recently successfully competed with traditional methods of material separation. The opportunity to cut intricate shapes [1,2] a diverse array of materials [3,4], including those that are difficult to machine [5,6], and the potential to carry out the process in harsh environments [7,8] (failure by fire or explosion, underwater operation, etc.) are the main reasons for this cutting technology’s versatility.

The application of this process is predominantly attractive for difficult-to-machine materials such as hardened steels [5,9,10], titanium alloys [6,11,12], nickel superalloys, and even tungsten alloys [13] or metal-matrix and polymer-matrix composites [14,15], ceramics [16,17], and ultra-high-strength steels, which are prone to cracking, heat-affected zones, or tool wear when processed by conventional methods. In comparison with common separation methods, this innovative production technology is characterized by a greater number of process parameters.

The material removal mechanism in AWJ combines three main erosion modes: micro-cutting, plastic deformation/plowing, and intergranular cracking/brittle fracture, whose relative contribution depends on the target material’s ductility, hardness, and fracture toughness [18,19,20]. In the upper zone of the kerf at shallow depth, the AWJ retains most of its kinetic energy and removes material primarily by microcutting, creating relatively smooth surfaces. As the jet penetrates deeper, it loses cohesion and kinetic energy due to viscous drag, abrasive particle fragmentation, and jet dispersion by the air. This leads to a gradual transition to deformational wear and fatigue-dominated erosion at greater depths, which manifests itself macroscopically as distinct curved striations (machining marks) and increasing surface roughness with depth [21,22,23].

Pressure increases jet velocity and thus kinetic energy of both water and abrasives, causing a higher material removal rate and deeper penetration, but also faster focusing-tube wear and higher energy consumption [24,25].

A higher abrasive mass flow rate supplies more cutting edges per unit time and causes increased depth of cut and reduced striation (footprint) angle up to an optimum. Beyond this point, further increases in abrasive flow yield a drop in returns and may even deteriorate surface quality due to particle interference and jet destabilization [26,27,28].

Traverse speed directly controls specific energy input per unit length. Low traverse speeds produce smooth, almost striation-free surfaces (Q5–Q4 quality classes [29]) but low productivity. High speeds dramatically increase the striation (footprint) angle, surface roughness, and kerf taper while reducing the depth of cut [30,31,32].

Therefore, modeling and a contemporary optimization approach are necessary to achieve excellent results [32]. Different statistical and mathematical techniques [33], metaheuristic approaches [34,35], methods that aid in decision-making [27,36,37], and even AI methods [38,39,40] and finite element methodology [41] were employed for this aim.

Dahil et al. [42] presented the most advantageous cutting method for high hardness, high strength, and superior toughness of Hardox 500 steel cutting. The authors presented the comparison of cuts by plasma, laser, wire erosion, and abrasive water jet (AWJ) methods. Based on microstructure photos of the cut surface made by different methods; the effects on the material’s metallurgical structure were compared.

Surfaces cut by the AWJ, because no thermal change did not occur on the jet contact area with the material, did not have any microstructure change or hardness differences, as in the other tested cutting. The cutting performance of multipass abrasive waterjet (AWJ) machining on Hardox steel was presented by Dekster et al. [10]. Research has shown that the right cutting parameters can result in superior performance with multipass cutting compared to single-pass cutting. It was shown that multipass cutting is rather efficient in increasing the depth of penetration and reducing the kerf taper angle, as it achieves 3 to 9 times higher depths.

Krenicky et al. [43] investigated how important control factors affected the surface quality of abrasive water jet (AWJ) cuts produced in Hardox (TM). Unfortunately, the equations were only created for measurements in one profile—the Ra and Rz roughness parameters—after the regression technique was applied to the collected data. To check the relations between Ra and Rz and the cutting parameters of pressure, traverse speed, and abrasive mass flow rate, regression equations were created. Regression equations describing correlations between the declination angle in the kerf as the independent variable, and the authors also provided either the Ra or the Rz parameters as dependent variables. Surface quality and cutting efficiency may be predicted using these models.

The surface quality of AWJ-cut parts is crucial in determining the cut components’ functional performance [44]. In this article, the impact of the three most significant control parameters—traverse speed, operating pressure, and abrasive flow—on one easy-to-observe factor, the jet deflection angle, was examined. It can be used to assess the treatment’s efficiency and quality in real time.

To determine the titanium surface profiles eroded by an abrasive waterjet (AWJ), Yuan Yemin et al. [45] created a surface profile evolution model that suppressed the AWJ’s eroding capacity and considered the jet’s directional deflection. Nonetheless, the findings showed that the roughness contours described by the surface development model agreed with the actual contours with a significant error of 11.4%.

Wala et al. [46] suggested comparing the chosen signal features of the cutting factors, which control the process course, and the parameters associated with the machined surface produced. The feed force recorded by the cutting process served as the basis for the AWJ deflection angle model.

Meanwhile, Brandstätter et al. [47] investigated the relationship between surface quality and the abrasive waterjet material disintegration parameters associated with machining marks. Materials with a wide range of hardness values were examined, such as aluminum, steel, and polyethylene plastic. The experiments were used to determine surface quality to assign a range of surface roughness parameters R-z and R-a when distinct machining marks cannot be recorded on the surface of specific materials with specific mechanical properties.

In the Lebar et al. paper [48], the possibility of using the thermovision camera or, for cutting transparent targets, an optical camera to examine the propagation of the AWJ cutting front was investigated. The characteristics of the cutting front can be extracted from the resulting photographs using digital image processing techniques, enabling process control.

The characteristic curved striations visible on AWJ-cut surfaces are a direct consequence of unsteady cutting front propagation and jet deflection toward the feed direction. The local striation (or footprint) angle δ (sometimes called the “declination” or “lag” angle) has been shown to correlate strongly with both instantaneous material removal capability and final surface roughness [43,49]. A low *λ* (≤10–15°) indicates stable, high-energy cutting conditions and typically corresponds to Ra < 3–5 µm on steels, whereas *λ* > 25–30° signals severe jet energy depletion and rough surfaces (Ra > 10–15 µm).

Despite extensive literature on empirical modeling of depth, roughness, and kerf taper [32,35,36], relatively few studies have systematically exploited the striation/footprint angle itself as a real-time diagnostic and control variable. Hashish [21] and later Hlaváček et al. [50] suggested that the instantaneous jet deflection angle can be optically monitored during cutting, offering a simple, non-contact method for adaptive control. Recent works using high-speed imaging and thermography [38,48] confirmed the feasibility of such approaches, yet robust quantitative models linking the three main parameters (pressure, abrasive flow rate, and traverse speed) directly to the measurable footprint angle *λ* are still scarce, especially for ultra-high-strength wear-resistant steels such as Hardox 500.

The present study addresses this gap by investigating the influence of pressure (350–400 MPa), abrasive mass flow rate (250–450 g/min), and traverse speed (100–300 mm/min) on the footprint (jet deflection) angle *λ* during AWJ cutting of 20 mm thick Hardox 500 steel. Second-order polynomial response models were developed for cutting depth, 3D surface roughness *Sq*, and—most importantly—the footprint angle *λ*. The practical objective is to demonstrate that online measurement of *λ* can serve as a single, easy-to-acquire indicator for simultaneous control of productivity and surface quality, even for new or inhomogeneous materials not covered by the machine’s built-in technological database.

Based on the above state-of-the-art analysis, it was observed that the surface quality of parts cut using the AWJ method, particularly the angle of the cutting marks (jet deflection angle), is crucial for determining the functionality of the cut parts.

This paper focuses on the most influential control parameters, such as feed rate, operating pressure, and abrasive mass flow rate, that affect the jet deflection angle. The objective is to determine the feasibility of online control of the AWJ cutting process by measuring the jet deflection angle. To achieve this, it was decided to establish the jet deflection angle based on the machining marks left behind. Further research will be planned to develop this method using optical methods and online digital image processing.

## 2. Materials and Methods

### 2.1. Cutting Material

In the research, Hardox 500 steel, characterized by an average hardness of 500 HBW, produced by the SSAB company (Stockholm, Sweden), was used. Hardox 500 maintains high toughness despite its high hardness, which is a key differentiator from conventional wear steels. The significant properties of Hardox 500 were presented in Table 1.

The chemical composition of Hardox 500 steel is presented in Table 2.

### 2.2. Abrasive Material

The most common use for almandine garnet is in AWJ technology. Like other garnets, almandine/Fe_3_Al_2_(SiO_4_)_3_/is an iron-aluminum garnet that forms spherical crystals with 12 rhombic or 24 trapezoidal faces, or combinations of these shapes, as presented in Table 3.

Garnet grains are acceptable for recycling up to five times without noticeable loss of performance [52]. Using first-time recycled abrasive grains can boost the cut efficiency [53], mainly due to good wear resistance and slight friability. Used garnet abrasive grains can be disposed of affordably and sustainably. Otherwise, they must be treated as industrial trash and exported to landfills.

Almandine Garnet type J80A from China (Jiangsu deposit) was used in the research [54].

Specific properties and chemical composition of this abrasive are presented in Table 4 and Table 5.

**Figure 1:**
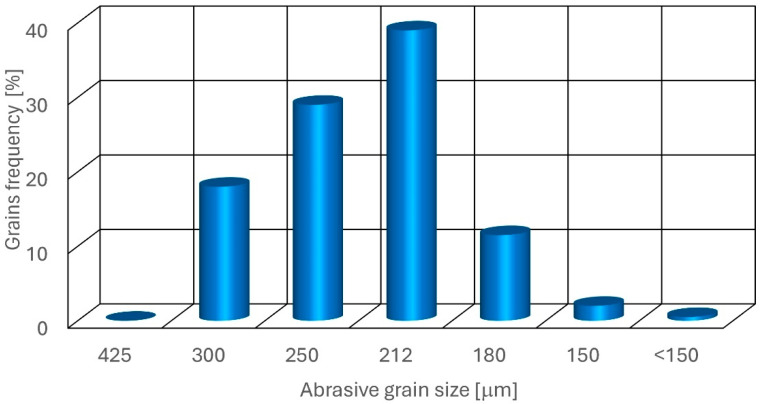
J80A almandine garnet grain distribution.

The grain size distribution (GSD) for this abrasive, shown in Figure 1, is typically characterized by an 80-mesh size, corresponding to a particle size range of 0.177 mm to 0.210 mm (177–210 μm) with a near-normal distribution.

This GSD is engineered to ensure optimal cutting performance in waterjet systems. The uniformity and consistency of the grain sizes are crucial for maintaining steady abrasive flow and achieving precise cutting edges. The absence of oversized grains prevents clogging in the focusing tubes, and the lack of undersized grains minimizes dust generation, enhancing both cutting efficiency and operator safety.

### 2.3. Test Rig

All research was carried out on the rig, built on the base of the precise abrasive waterjet machining center, the OMAX 60,120 (OMAX Waterjet, Kent, WA, USA). It offers the accurate, repeatable cutting of almost any material by combining high-speed water jet technology with the rigid bridge-style gantry system and a digital control system.

### 2.4. Design of Experiment (DoE)

A full factorial design of experiment matrix model with three process factors—pressure, abrasive flow rate (AFR), and traverse speed—was used. Factors were chosen based on previous scientific papers [55,56] and other articles [50,57,58].

The twenty-seven tests were carried out, according to the L27 array based on the Design of Experiment (DoE). Three levels of the following three control parameters were chosen as follows:

Abrasive Flow Rate: 250, 350, 450 [g/s],

Pressure: 350, 375, 400 [MPa],

Traverse speed: 100, 200, 300 [mm/min].

The following output parameters were selected:

- Cutting depth,
- Roughness of the cut surface *Sq*,
- Jet deflection angle.

### 2.5. Measurements

An Olympus DSX1000 optical microscope (Olympus, Tokyo, Japan), which merges remarkable precision and optical performance with smart measuring tools, was used to assess the roughness and the footprint angle of the cut groove’s lateral surface. The angular accuracy in practice, after calibration, is typically ±0.05–±0.2° for angles in the range 0–90°, depending on lighting conditions and surface properties. The accuracy of linear measurements is

- XY: up to 0.01 μm (subpixel interpolation in PRECiV),
- Z: up to 0.1 μm (in 3D mode with autofocus or scanning).

The outcomes of the two output parameters are displayed in Table 6:

The *Sq* parameter was selected to evaluate the cutting quality. *Sq* provides a statistical measure of surface roughness and highlights the distribution of surface heights. It is useful when recognizing surfaces with different topographical distributions but similar Sa values. *Sq* is calculated based on the following equation:

(1) $$Sq=\sqrt{\frac{1}{A}\iint_{A}Z^{2}(x,y)dxdy}$$

where

*A* is measuring area,
*x* and *y* are linear dimensions of an area.

The *Sq* factor is most useful for complex surface texture and is therefore preferred in high-precision fields where surface interactions are essential. *Sq* is more appropriate for applications where surface texture details are significant and could impact performance. Additionally, it is useful for assessing surfaces subject to specific interactions, e.g., with fluids or intense tribological effects.

**Figure 2:**
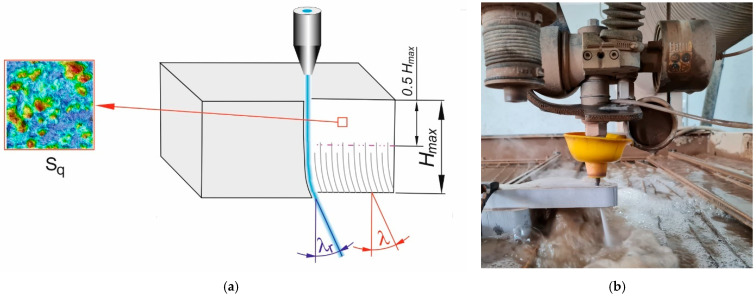
Measured output parameters: (**a**) schematic, (**b**) real view.

The *Sq* surface roughness (root mean square height) coefficient is often specified at the lowest value. Half the practical depth of cut, or roughly half the maximum depth of cut Hmax, was chosen as the site for the roughness measurement region (Figure 2). The workspace measured 0.977 mm by 0.977 mm. The measurement signals were processed using a Gaussian band-pass filter in accordance with ISO 25178-3 [59] to separate macro-geometry (form, waviness) from intrinsic roughness. The following measurement parameters were adopted for the tests:

S-Filter: λs = 2.5 µm

L-Filter: λc = 0.08 mm (cut-off)

The *λ* footprint angle characterizes cut efficiency and quality, requiring a minimal value. To eliminate the potential errors across the whole sample, footprint angles were measured five times for different footprints on each surface, and then the average value was considered.

## 3. Results and Discussion

Control parameters, their values, and output values are listed in Table 6.

### 3.1. Cutting Depth

Cutting depth is one of the criteria for evaluating the efficiency of AWJ machining, commonly used in many scientific and research centers [60,61]. The maximum cutting depth was used in this research due to the unambiguous evaluation. Based on test results (Table 5), the polynomial model of process control parameters’ influence on cutting depth was created:

*H*(*x*_1_,*x*_2_,*x*_3_) = 203.7 − 0.022*x*_1_ − 0.906*x*_2_ − 0.181*x*_3_ + 0.000023*x*_1_^2^ + 0.0012*x*_2_^2^ + 0.00021*x*_3_^2^ + 0.00002*x*_1_*·x*_2_ + 0.000018*x*_1_*·x*_3_ + 0.0001*x*_2_*·x*_3_,· (2)

where

*H* is cutting depth [mm],
*x*_1_ is abrasive flow rate [g/min],
*x*_2_ is pressure [MPa],
*x*_3_ is the traverse speed [mm/min].

**Figure 3:**
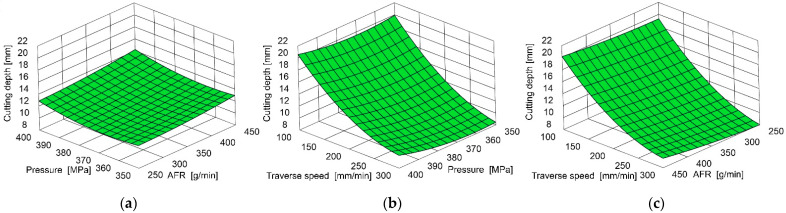
Example influence of AFR, pressure, and traverse speed on cutting depth at the following conditions: (**a**) 200 mm/min traverse speed, (**b**) 350 g/min AFR, (**c**) 375 MPa pressure.

An example graphical illustration of this equation is presented in Figure 3.

The strong dependence of cutting depth on traverse speed is evident, as the lowest feed rate achieved relatively high cutting depth under all other conditions.

The pressure has a second-order effect. High-pressure increases result in a decrease in the cutting depth in the tested area, most noticeable at the highest traverse speed.

Among the tested control parameters, the abrasive mass flow rate (AFR) per cut depth achieved the lowest result. Noteworthy is the relatively small relationship between cut depth and AFR for the lowest feed rates tested.

Table 7 shows the analysis of variance of the cutting depth.

### 3.2. Surface Roughness

A cut surface roughness is one of the criteria for assessing the quality of AWJ machining [62,63]. The roughness measure *Sq* was utilized in the research, which is, in fact, the root mean square value of the ordinate values in the defining area. It is comparable to the height standard deviation and recommended for determining the surface after AWJ cutting [64,65].

Based on research, the polynomial model of process control parameters’ influence on surface roughness was created:

*S*(*x*_1_,*x*_2_,*x*_3_) = 30.5 − 0.04*x*_1_−0.112*x*_2_ + 0.0495*x*_3_ + 0.000029*x*_1_^2^ + 0.00012*x*_2_^2^ − 0.00003*x*_3_^2^ + 0.000053*x*_1_*·x*_2_ − 0.000049*x*_1_*·x*_3_ − 0.00002*x*_2_·*x*_3_*x*_3_ (3)

where

*S* is the surface roughness factor *Sq* [μm],
*x*_1_ is abrasive flow rate [g/min],
*x*_2_ is pressure [MPa],
*x*_3_ is the traverse speed [mm/min].

**Figure 4:**
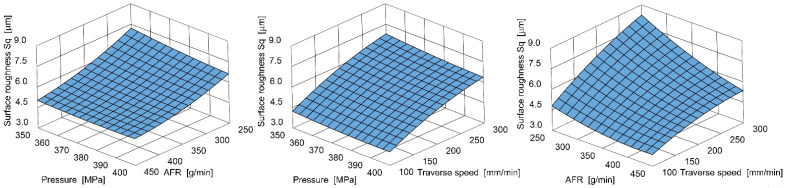
Example influence of AFR, pressure, and traverse speed on surface roughness.

This equation is graphically illustrated in Figure 4.

Additionally, in this case, the strong dependency of cut surface roughness on the traverse speed is evident. However, the abrasive flow rate (AFR) has a secondary influence here, while pressure has the least influence among the tested control parameters.

Table 8 shows the analysis of variance of the *Sq* cutting surface roughness.

### 3.3. Footprint Angle

AWJ effects were reflected on the machined surface in the curvilinear machining footprint forms and were measured to determine the jet deflection angle, as shown in Figure 2.

Based on research, the polynomial model of process control parameters’ influence on the deflection angle was created:

*D(x*_1_,*x*_2_,*x*_3_) = 167 − 0.022*x*_1_ − 0.962*x*_2_ + 0.3513*x*_3_ + 0.00006*x*_1_^2^ + 0.0014*x*_2_^2^ + 0.00021*x*_3_^2^ − 0.000006*x*_1_*·x*_2_ − 0.00024*x*_1_*·x*_3_ − 0.00068*x*_2_·*x*_3_ (4)

where

*D* is the deflection angle [deg],
*x*_1_ is abrasive flow rate [g/min],
*x*_2_ is pressure [MPa],
*x*_3_ is the traverse speed [mm/min].

**Figure 5:**
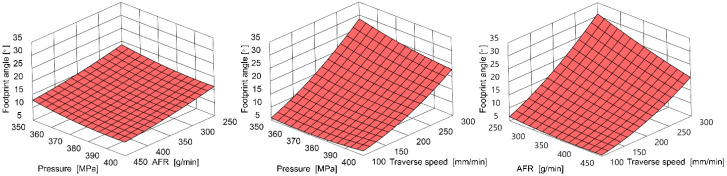
Example influence of AFR, pressure, and traverse speed on footprint angle.

An example illustration of this equation is shown in Figure 5.

The strong deviation angle dependence on the feed rate is visible. However, the abrasive flow rate (AFR) and pressure have a secondary order of influence among the tested control parameters.

Table 9 shows analysis of variance of the λ footprint angle.

**Figure 6:**
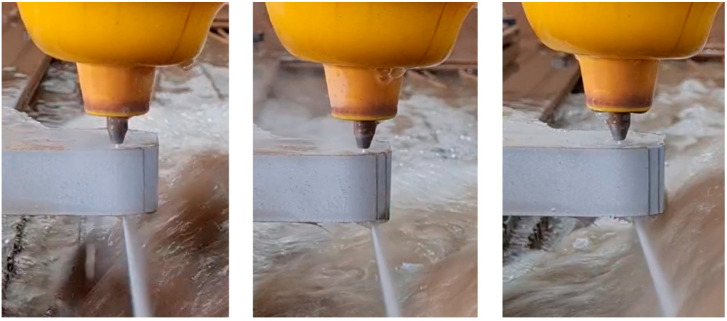
Example view of AWJ cutting process with different deflection angles.

**Figure 7:**
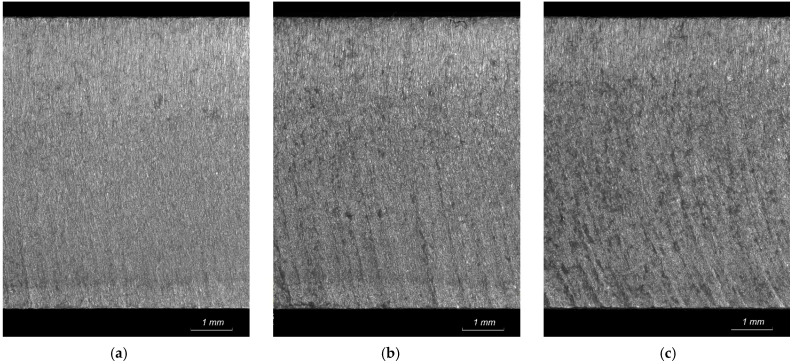
Example of the AWJ cutting surface with different footprint angle at the following traverse speed: (**a**) 100 mm/min, (**b**) 200 mm/min, (**c**) 300 mm/min.

Figure 6 shows examples of various l-angles throughout the cutting process. Figure 7 illustrates their relation to the machining footprints on the surface cut. The *λ* angle increases as the feed rate increases, and the larger curvature of the machining imprints confirms these observations.

### 3.4. Image Analysis to Support the AWJ Parameters Optimization

The parameters of the AWJ process, such as the pressure, cutting speed, nozzle stand-off distance, etc., are monitored separately by the process controller. In typical situations, the required cutting precision is achieved by tuning experimental process parameters. This leads to potential processing precision inaccuracy, as the real process is performed in environmental conditions and process-specified parameter uncertainty and instability.

The physical parameters of the process are expected to vary over time, and the control process usually assumes that the variations are of such a magnitude that they can be accepted within the tolerance of processing accuracy. The human-based control parameter selections are usually completed when an acceptable result is obtained under time-specific conditions.

Image analysis techniques are one of the controlling concepts of AWJ processing quality and overall performance. Some examples of image analysis applications are described in the literature, including

- support in the process of removal of organic coatings with rotating water jets, and the image analysis was applied to identify exposed steel areas [66],
- identification of cutting regions by detecting glass materials in AWJ-based CNC processes [67],
- identification of geometrical features of holes trepanned with AWJ [68] to assess the processing precision,
- identification of AWJ processing burr areas that require additional finishing [69],
- observation of cutting front parameters with thermal or optical cameras [48].

In general, the AWJ process can be supported by an image analysis system to improve operation accuracy, energy and material usage optimization, process time effectiveness, or other factors. The most desired effect of the operation of such a system should be the multicriteria system processing time improvement, cost, energy, and material effectiveness.

**Figure 8:**
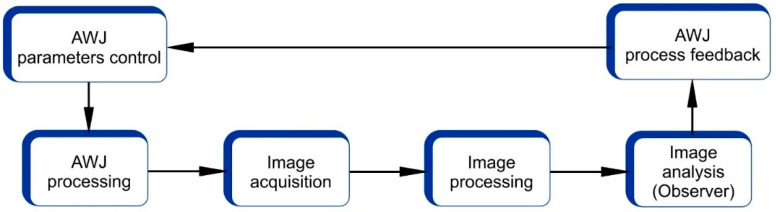
Proposed structure of the image analysis system to support the AWJ parameters optimization.

The structure of such a system would comprise an image acquisition module, an image processing module, a parameter observer module, and a process feedback module (Figure 8).

## 4. Conclusions

Grounded on the research, mathematical models of the machining process were established, showing the cutting depth, surface roughness, and jet deflection angle. For this purpose, second-degree polynomial equations were used, with three variables representing the control parameters of the AWJ cutting process.

The ability to evaluate the jet deflection angle was determined. This demonstrated the potential for correcting actual machining conditions to achieve the required throughput while maintaining the intended surface quality.

Measuring the deflection angle during the cutting process confirmed its control possibility, enabling steering of process efficiency while maintaining the intended cut surface quality.

Deflection angle measurement is another relatively simple alternative to the cutting process status control and to monitoring its quality and efficiency.

Further research will examine the technical feasibility of measuring the jet deflection angle using real-time digital optical analysis systems.

This will involve studying image acquisition during cutting, followed by image processing and analysis.

The precise relationship between the jet deflection angle and the condition of the cut surface will be investigated.

In the practical aspect of the cutting process, observing the jet deflection angle will allow the determination of process stability, especially when cutting natural materials characterized by property variability, such as rock materials.

## Figures and Tables

**Table 1:** Hardox 500 mechanical and physical properties.

Property	Value
Hardness	~470–530 HBW (Brinell)
Yield Strength	≥1000 MPa
Tensile Strength	~1250 MPa
Impact Toughness	27 J @ −40 °C (typical)
Density	7.85 g/cm^3^
Thermic Conductivity	~40 W/m·K
Specific Heat	~480 J/kg·K

**Table 2:** Hardox 500 chemical composition.

Element	C	Si	Mn	P	S	Cr	Mo	Ni
Contents [%]	0.38	0.7	1.7	≤0.02	≤0.01	1.2	0.65	1.0

**Table 3:** Properties of an almandine garnet [39,51].

Crystallographic System	Cubic
Unit cell	a = 11.53 Å
Break	Conchoidal to uneven
Color	Deep red, reddish-brown, brown to black, and deep brown.

**Table 4:** J80A almandine garnet properties.

Density	Hardness	Bulk Density	Melting Point
[kg/m^3^]	[Mohs]	[kg/m^3^]	[°C]
4100	7.5–8.0	1800–2100	1300

**Table 5:** J80A almandine garnet chemical composition.

SiO_2_	Al_2_O_3_	FeO	Fe_2_O_3_	MnO	TiO_2_	MgO	CaO	Free Silica
39.12%	20.92%	23.89%	4.15%	0.15%	0.10%	9.78%	9.56%	0%

**Table 6:** Control and output factors.

No	AFR [g/s]	Pressure [MPa]	Traverse Speed [mm/min]	Depth of Cut [mm]	Roughness Sq [μm]	Deflection Angle *λ* [°]
1	250	350	100	20.09	4.9430	8.58
2	250	350	200	10.74	7.1000	16.65
3	250	350	300	7.86	8.9420	36.74
4	250	375	100	19.79	4.9920	6.36
5	250	375	200	11.63	7.0100	15.67
6	250	375	300	8.64	8.0000	29.04
7	250	400	100	20.30	4.5270	8.52
8	250	400	200	12.89	6.7050	14.59
9	250	400	300	8.90	7.8610	30.85
10	350	350	100	20.21	4.2300	4.80
11	350	350	200	12.87	5.7000	13.63
12	350	350	300	8.37	6.9000	28.60
13	350	375	100	17.06	4.3470	7.32
14	350	375	200	12.30	5.3390	12.10
15	350	375	300	9.08	6.3060	27.13
16	350	400	100	21.06	3.8820	6.83
17	350	400	200	13.75	5.3710	14.68
18	350	400	300	9.78	6.7000	19.64
19	450	350	100	20.81	3.8140	6.33
20	450	350	200	12.76	5.2350	14.31
21	450	350	300	9.08	5.4290	22.41
22	450	375	100	20.80	3.8630	3.57
23	450	375	200	12.07	5.0649	13.07
24	450	375	300	9.96	5.1610	18.87
25	450	400	100	21.14	3.8770	5.56
26	450	400	200	14.53	4.8950	11.12
27	450	400	300	11.05	5.4160	18.18

**Table 7:** Analysis of Variance of Cutting Depth.

Source	DF	Adj SS	Adj MS	F-Value	*p*-Value
Model	9	582.965	64.774	97.22	0.000
Linear	3	552.875	184.292	276.62	0.000
ma	1	7.169	7.169	10.76	0.004
p	1	6.254	6.254	9.39	0.007
vp	1	539.452	539.452	809.70	0.000
Square	3	28.913	9.638	14.47	0.000
ma*ma	1	0.308	0.308	0.46	0.506
p*p	1	3.390	3.390	5.09	0.038
vp*vp	1	25.215	25.215	37.85	0.000
2-Way Interaction	3	1.177	0.392	0.59	0.631
ma*p	1	0.037	0.037	0.06	0.816
ma*vp	1	0.375	0.375	0.56	0.464
p*vp	1	0.765	0.765	1.15	0.299
Error	17	11.326	0.666		
Total	26	594.291			
Model summary:		R^2^ 98.09%	R^2^(adj) 97.09%	R^2^(pred) 95.99%	

DF—Degrees of Freedom, SS—Sum of Squares, MS—Mean Square.

**Table 8:** Analysis of variance of the *Sq* cutting surface roughness.

Source	DF	Adj SS	Adj MS	F-Value	*p*-Value
Model	9	48.9500	5.4389	119.74	0.000
Linear	3	44.6741	14.8914	327.84	0.000
ma	1	16.6755	16.6755	367.12	0.000
p	1	0.5199	0.5199	11.44	0.004
vp	1	27.4788	27.4788	604.96	0.000
Square	3	1.1427	0.3809	8.39	0.001
ma*ma	1	0.5172	0.5172	11.39	0.004
p*p	1	0.0343	0.0343	0.76	0.397
vp*vp	1	0.5911	0.5911	13.01	0.002
2-Way Interaction	3	3.1332	1.0444	22.99	0.000
ma*p	1	0.2139	0.2139	4.71	0.044
ma*vp	1	2.8900	2.8900	63.63	0.000
p*vp	1	0.0293	0.0293	0.65	0.433
Error	17	0.7722	0.0454		
Total	26	49.7222			
Model summary:		R^2^ 98.45%	R^2^(adj) 97.62%	R^2^(pred) 96.13%	

DF—Degrees of Freedom, SS—Sum of Squares, MS—Mean Square.

**Table 9:** Analysis of variance of the λ footprint angle.

Source	DF	Adj SS	Adj MS	F-Value	*p*-Value
Model	9	1998.97	222.11	56.06	0.000
Linear	3	1860.66	620.22	156.55	0.000
ma	1	159.49	159.49	40.26	0.000
p	1	27.08	27.08	6.84	0.018
vp	1	1674.08	1674.08	422.57	0.000
Square	3	33.13	11.04	2.79	0.072
ma*ma	1	2.22	2.22	0.56	0.464
p*p	1	4.60	4.60	1.16	0.296
vp*vp	1	26.31	26.31	6.64	0.020
2-Way Interaction	3	105.18	35.06	8.85	0.001
ma*p	1	0.00	0.00	0.00	0.979
ma*vp	1	70.91	70.91	17.90	0.001
p*vp	1	34.27	34.27	8.65	0.009
Error	17	67.35	3.96		
Total	26	2066.32			
Model summary:		R^2^ 96.74%	R^2^(adj) 95.02%	R^2^(pred) 91.59%	

DF—Degrees of Freedom, SS—Sum of Squares, MS—Mean Square.

## Data Availability

The original contributions presented in this study are included in the article. Further inquiries can be directed to the corresponding author.
